# PalmTraits 1.0, a species-level functional trait database of palms worldwide

**DOI:** 10.1038/s41597-019-0189-0

**Published:** 2019-09-24

**Authors:** W. Daniel Kissling, Henrik Balslev, William J. Baker, John Dransfield, Bastian Göldel, Jun Ying Lim, Renske E. Onstein, Jens-Christian Svenning

**Affiliations:** 1 Institute for Biodiversity and Ecosystem Dynamics (IBED), University of Amsterdam, P.O. Box 94240, 1090 GE Amsterdam, The Netherlands; 2 Section for Ecoinformatics & Biodiversity, Department of Bioscience, Ny Munkegade 114, DK-8000 Aarhus C, Denmark; 3 Royal Botanic Gardens, Kew, Richmond, Surrey TW9 3AE UK; 4 German Centre for Integrative Biodiversity Research (iDiv) Halle-Jena-Leipzig, Deutscher Platz 5e, 04103 Leipzig, Germany; 5 Center for Biodiversity Dynamics in a Changing World (BIOCHANGE), Department of Bioscience, Ny Munkegade 114, DK-8000 Aarhus C, Denmark

**Keywords:** Biodiversity, Tropical ecology, Tropical ecology, Macroecology, Coevolution

## Abstract

Plant traits are critical to plant form and function —including growth, survival and reproduction— and therefore shape fundamental aspects of population and ecosystem dynamics as well as ecosystem services. Here, we present a global species-level compilation of key functional traits for palms (Arecaceae), a plant family with keystone importance in tropical and subtropical ecosystems. We derived measurements of essential functional traits for all (>2500) palm species from key sources such as monographs, books, other scientific publications, as well as herbarium collections. This includes traits related to growth form, stems, armature, leaves and fruits. Although many species are still lacking trait information, the standardized and global coverage of the data set will be important for supporting future studies in tropical ecology, rainforest evolution, paleoecology, biogeography, macroecology, macroevolution, global change biology and conservation. Potential uses are comparative eco-evolutionary studies, ecological research on community dynamics, plant-animal interactions and ecosystem functioning, studies on plant-based ecosystem services, as well as conservation science concerned with the loss and restoration of functional diversity in a changing world.

## Abstract (summary)

**Table:** 

Measurement(s)	fruit length • stem length • plant height • petiole length • fruit color • leaf length • plant trait • fruit morphology trait
Technology Type(s)	digital curation
Factor Type(s)	species
Sample Characteristic - Organism	Arecaceae
Sample Characteristic - Environment	tropical lowland evergreen broadleaf rain forest • tropical moist broadleaf forest biome • tropical deciduous broadleaf forest • tropical woodland biome • subtropical woodland biome
Sample Characteristic - Location	global

Machine-accessible metadata file describing the reported data: 10.6084/m9.figshare.9766919

## Background & Summary

Most ecosystems are composed of a large number of species with different characteristics. These characteristics (i.e. traits) reflect morphological, reproductive, physiological, phenological, or behavioural measurements of species that are usually collected to study intraspecific trait variation (i.e. among individuals or populations of the same species) or interspecific trait variation (i.e. among species)^1–5^. Many traits have an important functional role for species and ecosystems and are therefore referred to as ‘functional traits’. For instance, functional traits such as plant morphological and physiological properties are often directly linked to ecosystem structure and ecosystem functioning^6,7^. Such functional traits are further important for the response of organisms to their environment (‘response traits’) and the effects of organisms on ecosystems and other species (‘effect traits’)^2,6,8^. Hence, functional traits are key to understanding ecosystem dynamics and the response of organisms to human-induced disturbances and changing environmental conditions such as climate change^4,9,10^, habitat fragmentation^11^ or harvesting pressure^12^.

Over the last few years, comprehensive trait databases with continental or global scope have become available, covering diverse taxa in the marine^13,14^ and freshwater realm^15^ as well as terrestrial taxa such as plants^16^ and vertebrates^17–20^. Despite these monumental efforts that have involved community contributions as well as advanced techniques in data mining and data integration, digitally available information on functional traits is still missing for the majority of taxa on Earth^3,21^. Even for well-studied organisms such as vascular plants, information remains taxonomically and geographically limited. For instance, the TRY plant trait database^16^ has achieved an impressive compilation of almost 12 million trait records for currently 280,000 plant species (TRY database version 5 released in March 2019, www.try-db.org), but often only a few trait records are available per species. Moreover, as for other ecological information such as species occurrences^22^, digitally accessible information on traits remains particularly scarce in the tropics where most biodiversity occurs^16,23–25^. This is a major bottleneck for ecological and evolutionary science because tropical ecosystems such as rainforests are one of Earth’s greatest biological treasures, a major source of ecosystem services for a large proportion of the global human population, and a key component of the Earth system^26,27^.

In the tropics, palms are an iconic plant family with keystone importance in many forest and savanna ecosystems^28–30^. The pantropical palm family (Arecaceae or Palmae) is species-rich and contains nearly 2600 species in 181 genera and 5 subfamilies^31^. The ecology and evolution of palms is strongly linked to interspecific variation in growth, reproduction and morphology of stems, leaves, inflorescences, fruits and seeds^32^. Palms are a major resource for herbivores, pollinators and fruit- as well as seed-eating animals in the tropics^29,30,32–34^, provide provisioning services such as food, construction material and medicine to people (especially in rural communities)^35^, and belong to one of the most economically important plant groups globally^36^. Moreover, palms can provide important insights into the evolution of tropical rainforests^28,37–39^, historical biogeography^40–42^, past climate change^43–45^ and the vulnerability and response of ecosystems to ongoing and future global change^46–48^. Despite this outstanding role of palms in tropical ecosystems and tropical biological science, studies using palm functional trait data across broad spatial scales remain scarce^35,38,49,50^.

Here, we introduce the PalmTraits 1.0 database, an extensive database containing functional traits for palm species globally. PalmTraits 1.0 releases information on error-checked and referenced traits to capture interspecific variation in growth forms, armature and the morphology of stems, leaves and fruits of palms. Species-level trait information was assembled from >130 sources including monographs and taxonomic revisions as well as credible online sources and two herbaria with extensive palm collections. By making these data available to the scientific community, we aim to advance the sharing and digitalization of ecological trait data and understanding of the global ecology, biogeography and evolution of palms and the tropical rainforests they inhabit.

## Methods

**Fig. 1:**
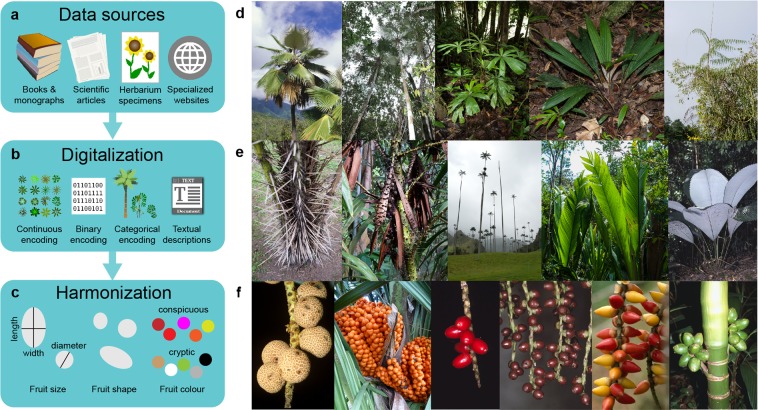
Trait compilation and trait variation in palms. The workflow (**a**–**c**) illustrates key steps in the compilation of palm trait data whereas the images (**d**–**f**) represent examples of trait variation in palms. (**a**) Main data sources for extracting trait data for PalmTraits 1.0. (**b**) Digitalization of the original trait information through different ways of encoding. (**c**) Standardization and harmonization of fruit trait information (size, shape and colour). (**d**) Palm growth forms (from left to right): *Pritchardia viscosa*, an erect canopy palm from Hawaii. Two erect palms (*Thrinax radiata* and *Coccothrinax argentata*) growing under a canopy gap in Mexico. *Licuala telifera*, an understory palm from New Guinea. *Dypsis acaulis*, an acaulescent understory palm from Madagascar. *Plectocomiopsis geminiflora*, a climbing rattan palm from Borneo. (**e**) Stem and leaf variation (from left to right): *Astrocaryum standleyanum* from Colombia, armed with long black spines. *Daemonorops didymophylla* from Southeast Asia, a climbing rattan palm armed with spines on the petiole of the leaf. *Ceroxylon quindiuense* from Colombia, with about 60 m stem height the tallest non-climbing palm in the world. *Marojejya darianii* from Madagascar, a medium-sized tree palm with large leaves of up to 5 m bade length. *Johannesteijsmannia magnifica* from Malaysia, an acaulescent palm with up to 2 metres long leaf blades covered with fine white hairs. (**f**) Fruit variation (from left to right): *Lemurophoenix halleuxii* from Madagascar, a canopy palm with large (5 cm) chestnut-brown fruits that have corky warts. *Ravenea dransfieldii* from Madagascar, a mid-story palm with small (1.5–2 cm) orange fruits. *Calyptrocalyx* sp., representing a genus of predominantly understory palms that have mostly small (1–2 cm) bright red fruits. *Hydriastele microspadix* from New Guinea, a mid-story palm with small dark red fruits. *Drymophloeus litigiosus* from New Guinea, an understory palm with small (1 cm) yellow to red fruits. *Areca ipot* from the Philippines, with large (5 cm long) fruits that ripen from green through yellow to red. Image credits: J. Dransfield, H. Balslev, and W.J. Baker.

The data collection of the PalmTraits 1.0 database involved three major steps (Fig. 1a–c): (1) the identification of data sources, (2) the digitalization and encoding of trait values, and (3) the harmonization of fruit size data. Overall, the database was designed to capture species-level (interspecific) trait variation of palms rather than individual-level (intraspecific) variability. Such aggregated data (e.g. average values of continuous traits) facilitate biodiversity data integration across large spatial, temporal, and taxonomic scales, but are limited in their capacity to resolve fine-grained ecological patterns^51^. The PalmTraits 1.0 database captures trait variation of palms in terms of growth forms, stems, armature, leaves, and fruits (Online-only Table 1). This represents a large variation of trait diversity in palms (Fig. 1d–f). Some fundamental traits like wood density, specific leaf area or N-content^5^ are not represented because these traits are not commonly measured by palm taxonomists and hence are not available from palm books, monographs, species descriptions or herbarium specimens. Nevertheless, some of the available traits reflect major dimensions of plant form and function^5^, including the size of whole plants (e.g. growth form and stem height) and their organs (e.g. blade length). Other traits also capture characteristics that are relevant for studying plant-animal interactions such as herbivory (e.g. stem and leaf armature)^48^ and frugivory and animal-mediated seed dispersal (e.g. fruit length and width, fruit shape, and fruit colour)^37,50,52^. Below we describe the data collection (Fig. 1a–c) in more detail.

### Data sources

The main data sources for extracting the palm trait data were books and monographs, scientific articles (e.g. taxonomic revisions and species descriptions), herbarium specimen and specialized websites (Fig. 1a). We first extracted trait data from books, monographs and taxonomic revisions because these contain trait descriptions in a standardized way and for major clades or specific regions. We started the trait data extraction by obtaining maximum values for stem height, stem diameter, leaf number and fruit diameter as well as binary information (yes/no) for acaulescence and stem clustering for about 850–1250 species from the appendix I of the palm ecology and evolution book of A. Henderson^32^. We then extracted additional information for continuous traits (minimum, maximum and average values) as well as binary or categorical traits from books that synthesised species-specific palm knowledge for particular countries or regions (e.g. Africa^53–56^, Americas^57^, Australia^58^, Brazil^59^, Colombia^60^, Costa Rica^61^, Ecuador^62^, Hawaii^63^, Indonesia^64^, Madagascar^65–67^, Malaysia^68,69^, Mascarene Islands^70^, New Caledonia^71^, Philippines^72^, Sabah^73^, Southern Asia^74^, Thailand^75^, Vietnam^76^). Additionally, we went through taxonomic revisions, monographs and other publications that provided trait data for specific taxonomic groups such as palm genera or tribes (e.g. *Acrocomia*^77^, *Aiphanes*^78^, *Archontophoenix*^79^, *Areca*^80^, *Asterogyne*^81^, *Astrocaryum*^82,83^, *Attalea*^84^, *Bactris*^85,86^, *Balaka*^87^, *Borassodendron*^88^, *Butia*^89^, *Calyptrocalyx*^90^, *Calyptrogyne*^91^, *Calamus*^92–94^, *Caryota*^95,96^, *Chamaedorea*^97–103^, *Cyrtostachys*^104^, *Drymophloeus*^105^, *Eremospatha*^55,106^, *Geonoma*^107^, *Heterospathe*^108^, *Hydriastele*^109,110^, *Hyospathe*^111^, *Johannesteijsmannia*^112^, *Laccosperma*^55,106^, *Lanonia*^113^, *Licuala*^114–116^, *Linospadix*^90,117^, *Livistona*^118,119^, *Metroxylon*^120^, *Nenga*^121^, *Oncocalamus*^55,106^, *Orania*^122^, *Parajubaea*^123^, *Phoenix*^124^, *Pinanga*^125^, *Ptychosperma*^126^, *Pritchardia*^127^, *Rhapis*^128^, *Sabal*^129^, *Syagrus*^130–134^, *Veitchia*^135^, *Wallichia*^136^). We further obtained raw data (i.e. individual-level trait measurements) from A. Henderson that were used in taxonomic revisions for a number of palm genera, including *Calyptrognye*^91^, *Chuniophoenix*^137^, *Desmoncus*^138^, *Geonoma*^107^, *Hyospathe*^139,140^, *Leopoldinia*^141^, *Pholidostachys*^142^, *Rhapis*^143^, *Synechanthus*^144^ and *Welfia*^145^. These raw data allowed us to add a few additional trait data (especially minimum, mean and maximum fruit sizes) for 139 species. We additionally used other scientific literature on palms^146–174^ as well as specialized palm websites^175–179^, and the book Genera Palmarum^180^ for traits that do not vary within single genera (e.g. some genera have only climbers). Finally, we visited two major herbaria (Aarhus University Herbarium, Denmark, and the Royal Botanic Gardens, Kew, UK) harbouring very large palm collections to fill gaps in the database by obtaining trait information from herbarium specimens (e.g. measuring fruit sizes or recording fruit colour from specimen descriptions). All sources are provided together with the trait dataset in DRYAD^181^.

### Digitalization

The digitalization of trait data from the original data sources into a database required to encode the information as continuous, binary, categorical or as text descriptions (Fig. 1b). For most traits, trait information was encoded either as continuous or binary (Online-only Table 1). For continuous traits, we usually recorded maximum values (e.g. for stem and leaf size) or minimum, maximum and average values (e.g. for fruit size) as reported in monographs and taxonomic revisions (Online-only Table 1). Binary traits were encoded as 0/1 (e.g. presence/absence of climbing, acaulescent or erect growth form, stem clustering, armature) and additionally as 2 if populations of the same species showed intraspecific trait variation (Online-only Table 1). Three traits were encoded as categorical information. This included understory/canopy information (a derived trait based on whether maximum stem height is ≤5 m or >5 m, and/or whether species have an acaulescent growth form or not)^50^, small/large fruit sizes (a derived trait based on whether fruit length is <4 cm or ≥4 cm in length, i.e. classifying small vs. megafaunal fruits)^50,182^, and fruit shape (Online-only Table 1). Two other traits (fruit colour and fruit shape) were encoded with text descriptions (Online-only Table 1). For those, we extracted verbatim text descriptions from the literature and herbarium sheets (e.g. glossy black, bright orange, or reddish brown as examples of fruit colour information) and later standardized and harmonized the information (see below).

### Harmonization

Since our research has a particular focus on palm-frugivore interactions^37,50,52,183^, we further standardized and harmonized trait information on fruit size, fruit shape, and fruit colour (Fig. 1c).

For fruit size, the PalmTraits 1.0 database provides information on average, minimum and maximum values for both fruit length and fruit width (Online-only Table 1). However, in some monographs, species descriptions and taxonomic revisions the original information on fruit size was reported as fruit diameter rather than fruit length and fruit width. This typically included palm species that tend to have roundish fruits. We initially recorded these fruit diameter measurements in a separate column, but then merged it into the fruit length and/or fruit width columns. There was a measurement difference between fruit diameter compared to fruit width and length estimates for 168 palm species. For 74 of those 168 species, this difference was ≤0.1 cm and we therefore ignored (deleted) fruit diameter information. For the remaining 94 species, we revisited the original sources and additionally checked available online sources. In 82 cases, fruit diameter/width/length did not differ much (0.1–0.5 cm), and we updated the fruit width information based on the fruit diameter measurements. In the 12 remaining cases, fruit diameter values were much smaller or larger than fruit length (difference >0.5 cm), and we decided to omit these fruit diameter values to avoid biases and outliers.

For fruit shape, we harmonized the original trait descriptions from the literature into seven categories (ellipsoid, elongate, fusiform, globose, ovoid, pyramidal, and rounded) (Online-only Table 1). We chose those categories as they were most widely used. Note that these fruit shape descriptions are not necessarily distinctively different because no quantitative formulas are used when taxonomists describe the fruits.

For fruit colour, we kept the extracted verbatim text descriptions from the literature (‘FruitColorDescription’ in Online-only Table 1), but we additionally aggregated and harmonized the verbatim text descriptions in two ways. First, we derived the main fruit colour(s) from the verbatim text descriptions (‘MainFruitColors’ in Online-only Table 1) and separated them by semicolons (e.g. ‘black; blue’, or ‘brown; orange; yellow’). This allowed to keep the main fruit colour descriptions, but simplified and reduced the verbatim text. Immature fruit colours were excluded in this step, and fruit colours described with a suffix -ish or –ey were usually reduced to the main fruit colours. Second, we classified fruit colours into ‘cryptic’ and ‘conspicuous’ colours (‘Conspicuousness’ in Online-only Table 1). This was done because fruit-eating animals can differ in their colour vision, for instance birds vs. bats or dichromatic vs. trichromatic primates^33^. We classified fruit colours as cryptic if their reflectance spectra are difficult to detect against a background of leaves, and as conspicuous if reflectance spectra appear to be in strong contrast to the background of leaves^184^. Consequently, orange, red, yellow, pink, crimson and scarlet fruits were classified as conspicuous, and brown, black, green, blue, cream, grey, ivory, straw-coloured, white and purple fruits as cryptic (following ref.^185^). When a fruit colour description contained a combination of cryptic and conspicuous colours (e.g. ‘green/yellow’, ‘yellow-brown’, ‘brown orange’), or when colours were described with a suffix -ish or –ey (indicating to have only a touch of that colour), we inferred that the cryptic colour is the dominant hue and the fruit colour was classified as cryptic. The classification of cryptic vs. conspicuous is here provided as an example to show how the verbatim text descriptions of fruit colours could be used for ecological or evolutionary analyses, e.g. when analysing the colour vision of primates in relation to the distribution of palms with conspicuous fruit colours. Other colour classifications can be developed from the colour verbatim text descriptions as originally extracted from the data sources (column ‘FruitColorDescription’, Online-only Table 1).

### Taxonomy

To standardize the taxonomic names of palms, we followed the World Checklist of palms^186^, using a version download from July 2015. This included a total of 2557 accepted palm species names. Since the palm taxonomy is regularly updated by taxonomic experts from the Royal Botanic Gardens in Kew, we recommend to use their taxonomic resources to search for synonyms and currently accepted names. Two useful online resources are the World Checklist of Selected Plant Families (WCSP, https://wcsp.science.kew.org, searching for ‘Arecaceae’) and PalmWeb (http://www.palmweb.org/).

## Data Records

The PalmTraits 1.0 database can be downloaded from the DRYAD data repository^181^ under the terms of a Creative Commons Zero (CC0) waiver. The CC0 waiver facilitates the discovery, re-use, and interoperability of the data by removing any legal barriers. We also provide the PalmTraits 1.0 database in the TRY Plant Trait Database (https://www.try-db.org/; TRY DatasetID 540) which uses a Creative Commons Attribution License (CC BY 4.0). Regardless of the database version used, we ask users to cite this data paper when these data are used in publications or other activities (e.g. teaching and education), and to also cite the actual version of the database used in accord with emerging standards for data citation.

### Data coverage

**Fig. 2:**
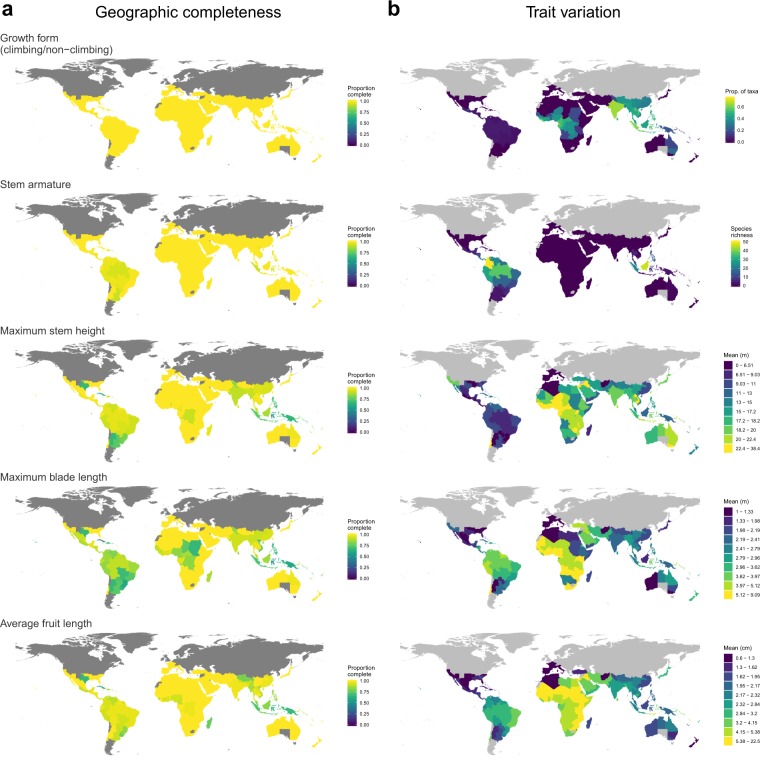
Geographic variation of trait information for palm assemblages worldwide. In (**a**), geographic completeness of various palm trait data is shown at the spatial resolution of ‘botanical countries’ (TDWG level 3 units) which are standardized areas defined by the International Working Group on Taxonomic Databases (TDWG) for recording plant distributions^193^. Global palm distribution data from the World Checklist of Palms are available as presence-absence data in TDWG level 3 units^186^ and can be used to analyse the global distribution and biogeography of palm assemblages^37,38,43,206^. Geographic completeness is represented here as the proportion of species having trait information, with yellow showing botanical countries with high completeness and dark blue showing botanical countries with low completeness. In (**b**), interspecific variation of traits is shown for palm assemblages in botanical countries. Trait variation is exemplified as the proportion of species having a specific growth form (e.g. proportion of climbers), as the species richness of palm species with a particular binary trait (e.g. stem armature), or by representing the mean value of a continuous trait (e.g. maximum stem height, maximum blade length, or average fruit length) across all palm species in a given botanical country. Yellow indicates botanical countries with high trait values and dark blue low trait values.

The PalmTraits 1.0 database covers 24 traits and additional taxonomic information (Online-only Table 1). The species coverage of trait information is complete for growth form (100% coverage), and particularly high for armature (>95%), stem habit (>84%), maximum stem height and diameter (>73%), and for average fruit size and width (>77%). Other traits are less covered (30–70%, Online-only Table 1), reflecting a lower availability of these traits in the published literature. Nevertheless, the high species coverage of several traits translates into a high geographic completeness of traits within country-level palm assemblages worldwide (Fig. 2, left column). For instance, global coverage of trait information is (near) complete for growth form and stem armature (Fig. 2, left top two maps). Other traits (e.g. maximum stem height, maximum blade length and average fruit length) have lower sampling completeness in species-rich tropical areas such as parts of South America, the Caribbean, Central Africa and Southeast Asia (Fig. 2, left bottom three maps).

**Fig. 3:**
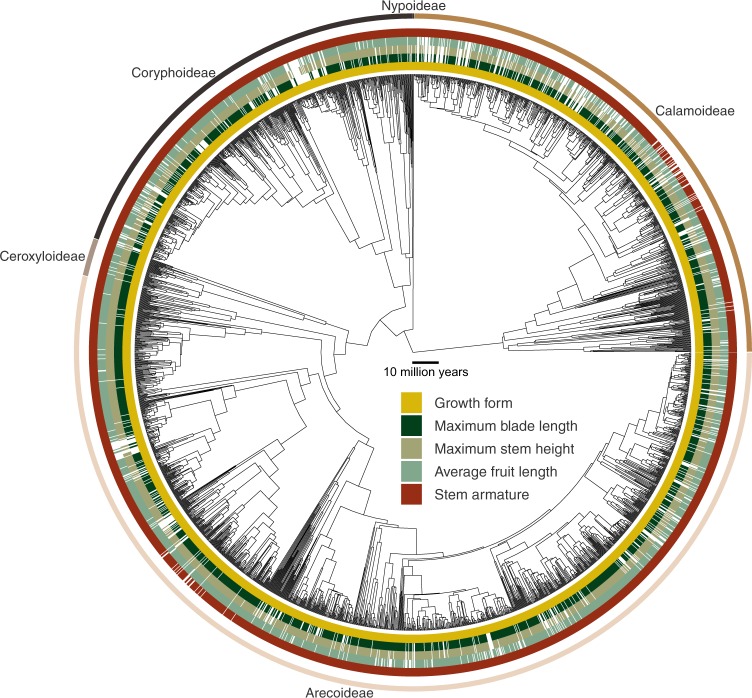
Phylogenetic distribution of exemplar palm traits. The five inner coloured circles represent species-level presence of trait information for key traits (growth form, maximum blade length, maximum stem height, average fruit length and stem armature). The outer coloured circle represents the five subfamilies of palms (Arecoideae, Ceroxyloideae, Coryphoideae, Nypoideae and Calamoideae). The time-calibrated phylogenetic tree illustrated here is a maximum clade credibility (MCC) tree^50,52^ derived from a recently published all-evidence species-level supertree of palms^187^.

Mapping species-level trait information to a phylogeny allows visualizing the phylogenetic coverage of traits. Using a recently published all-evidence species-level supertree of palms^187^, we demonstrate that little phylogenetic bias exists in the coverage of key traits across the palm family (Fig. 3).

### Applications

The PalmTraits 1.0 allows the analysis of trait variation within palm species assemblages worldwide (Fig. 2, right column). This includes mapping the predominance (i.e. proportion) of particular growth forms (e.g. climbers), the species richness of palms with particular traits (e.g. stem armature), or the average size of stems, leaves or fruits across species that are present within botanical countries (Fig. 2, right column). Another avenue of application is to combine the species-level trait information with phylogenies (e.g. the recently published all-evidence species-level supertree of palms^187^) to perform macroevolutionary analyses such as trait-dependent models of speciation, extinction and transition rates^50,52^.

## Technical Validation

All data were digitized by entering trait information from the original source (e.g. books, taxonomic revisions or specimen sheets) into an Excel spreadsheet, where each row represented a palm species and each column a single trait. Subsequent error detection and data quality control were done at three levels. First, trait information on growth forms (climbing, acaulescent, and erect) was carefully checked by a taxonomist (J.D.) with comprehensive experience with palms in the field and herbarium. Trait information of some specific palm genera was further checked by additional experts (see acknowledgements). Second, we sorted and filtered the Excel spreadsheet to search for erroneous entries (i.e. obvious errors in data entry) such as text or comma entries in columns with continuous data, or negative trait values and wrong values from unit conversion. These were corrected as much as possible. Third, we identified extreme values and detected outliers by looking at the most extreme (smallest and largest) values of each continuous trait across the whole family as well as within each tribe. These extreme values were checked for plausibility and reliability against external sources and our taxonomic and ecological knowledge of palms, and retained or corrected accordingly. For instance, several climbing palms (especially in the genus *Calamus*) have stem heights ≥100 m, with *Calamus manan* being the tallest climbing palm in the world (with 170 m stem height or more)^188^. Among erect palms, *Ceroxylon quindiuense* is with >60 m the tallest^189^. Fruit size is largest for *Lodoicea maldivica* (50 cm), the palm with the largest seed within the whole plant kingdom^190^. In contrast, the smallest fruit sizes are found in palm species in the genus *Coccothrinax*^191^. Palms also hold the record of the largest leaf of the plant kingdom, with *Raphia regalis* having a maximum blade length of 25 m^192^.

## Usage Notes

We provide the data via the Dryad digital repository^181^ and via the TRY plant trait database (www.try-db.org; TRY DatasetID 540). The Dryad release^181^ contains three files related to the PalmTraits 1.0 database:

1. A tab-delimited text file containing taxonomic information (species, genus, tribe, subfamily) together with all trait data
2. A tab-delimited text file containing all references that have been used for each species.
3. A tab-delimited text file containing the full details of all references that were used.
  To facilitate integration with other datasets, we further provide the following files (also via the Dryad data repository^181^):
4. An R script containing code that allows to combine the PalmTraits 1.0 database with species distribution and phylogenetic information
5. A shape file with all botanical countries (TDWG level 3 units) worldwide
6. Presence-absence data of palms at the resolution of botanical countries
7. Phylogenetic information of palms represented as maximum clade credibility (MCC) tree

### Tips for integrating the data records with other datasets

**Fig. 4:**
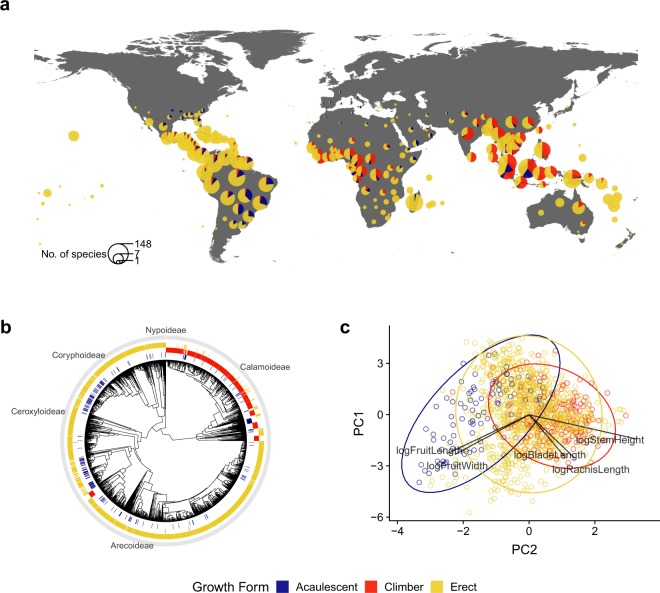
Examples of combining palm trait data, species distribution and phylogenetic information. The global map in (**a**) shows the relative proportion of major palm growth forms within ‘botanical countries’ worldwide (i.e. geographic units as defined by the International Working Group on Taxonomic Databases, TDWG^193^) by combining growth form information (climbing, acaulescent, and erect) with global species distribution data from the world checklist of palms^186^. In (**b**), palm growth form information is linked to a species-level palm phylogeny^187^ using a Maximum Clade Credibility (MCC) phylogenetic tree^50,52^ to illustrate the phylogenetic distribution of climbing, acaulescence and erect growth forms in palms. Climbing dominates in the subfamily Calamoideae whereas erect palms are common in subfamilies Coryphoideae, Ceroxyloideae and Arecoideae. Acaulescent palms are scattered across the palm phylogenetic tree. In (**c**), the location of different palm growth forms (climbing, acaulescent, and erect) in a multivariate trait space is illustrated by the first two axes of a Principal Component Analysis (PCA) based on continuous trait information on stem height (logStemHeight), leaf size (logBladeLength and logRachisLength) and fruit size of palms (logFruitLength and logFruitWidth). The figure can be reproduced with data and an R script that integrates the PalmTraits 1.0 database with spatial and phylogenetic data^181^.

The R script that we provide contains guidance of how to integrate the PalmTraits 1.0 database with spatial and phylogenetic data and how to explore multi-variate trait variation^181^. We illustrate this by using the growth form information (climbing, acaulescent, and erect) from PalmTraits 1.0 (Fig. 4). We first load global species distribution data from the world checklist of palms^186^ and then combine them with the new palm growth form data and a polygon file that represents geographic units (‘botanical countries’, i.e. TDWG level 3 units) as defined by the International Working Group on Taxonomic Databases (TDWG), a geographic standard for recording plant distributions^193^. This allows plotting the proportion of growth forms in palm assemblages worldwide (Fig. 4a). We then map growth form information onto a species-level palm phylogeny^187^ using a Maximum Clade Credibility (MCC) phylogenetic tree as recently used in macroevolutionary analyses of palms^50,52^. This allows to explore growth form information in a phylogenetic context (Fig. 4b). Finally, the R script illustrates how continuous trait information (e.g. on stem height, leaf size and fruit size) can be combined with growth form information to explore the multi-dimensional nature of species traits (Fig. 4c).

### Imputation of missing trait data

As trait values are often not available for all species (Online-only Table 1), we recommend to explore data imputation methods to fill information for missing data. Data imputation might be especially important for analyses where complete trait-based representation of all palm species is crucial. For instance, metrics of functional diversity^194^ can be systematically biased when trait data coverage is incomplete^195,196^ and gap-filling may allow to reduce errors when interpreting functional diversity patterns^197^. Data imputation may be performed in a variety of ways, for example through the leveraging of phylogenetic comparative models^198^, taxonomic hierarchies^199^, or machine learning algorithms^200^. The relative performance and accuracy of the methods will depend on completeness and interspecific and intraspecific variation of traits. For instance, if correlations among traits are not strong, predictions based on observed covariation in existing trait data should be used with caution. We suggest that data imputation methods should be rigorously tested and accompanied with comprehensive sensitivity analyses to assess their performance^201^.

### Semantic integration with other plant trait data

Plant trait data are measured in a multitude of ways^202^, and this heterogeneity together with a lack of standards for acquiring, organizing and describing trait data makes their integration often difficult^3,203,204^. Trait data of palms are usually described in a standardized and systematic way within taxonomic descriptions and revisions. This makes the extraction of palm trait data relatively straightforward. However, many of the palm trait terms and measurements are not directly captured in semantic descriptions of plant traits such as the global handbook for standardised measurement of plant functional traits^205^ or the thesaurus of plant characteristics (TOP)^203^. During the collection of palm trait data, we did not harmonize the terminology of palm trait definitions with other plant trait terminologies because they were internally consistent (i.e. within the palm family). However, after finalizing the data collection we mapped the palm trait definitions to the TOP (see Online-only Table 1). Several palm traits are currently not represented in the TOP. For instance, fruit colour is currently not represented within the dispersule trait category of the TOP. Similarly, maximum number of leaves as well as armature on leaves and stems are currently not captured by the TOP. This highlights the need for further development of the TOP and other semantic resources to facilitate the integration of trait data from multiple sources^3^. Such efforts will also allow better interoperability and effectiveness of automated data exchange among different sources. We therefore urge the research community to further develop and harmonize existing plant traits terminologies and semantic relations.

## Data Availability

The original data collection was done by entering trait information into an Excel spreadsheet (Microsoft Office 2013). No code is available for this step. The final PalmTraits 1.0 dataset was saved as tab-delimited text file^181^. Scripts to load the PalmTraits 1.0 dataset into R, to plot multi-variate trait variation and to combine it with phylogenetic and species distribution data are available in the accompanying dataset^181^. The scripts were developed in R version 3.5.0, and using the associated libraries as indicated in the scripts. There are no restrictions to use the provided code.

## Online-only Table

**Online-only Table 1:** Available trait information in PalmTraits 1.0.

Header	Description	TOP category	Type	Unit	Coverage (# species)	Coverage (% species)
**Taxonomy**						
SpecName	Taxonomic name of species (binomial nomenclature) following the World Checklist of palms^186^	—	Text	—	2557	100
accGenus	Accepted genus name from the World Checklist of palms^186^	—	Text	—	2557	100
accSpecies	Accepted species name from the World Checklist of palms^186^	—	Text	—	2557	100
PalmTribe	Name of palm tribe from the World Checklist of palms^186^	—	Text	—	2557	100
PalmSubfamily	Name of palm subfamily from the World Checklist of palms^186^	—	Text	—	2557	100
**Growth form and habit**						
Climbing	Whether palm species has climbing habit or not, or both if populations vary in this trait	whole plant growth form	Binary	Climbing (1), non-climbing (0), both (2)	2557	100
Acaulescent	Whether palm species has an acaulescent growth form (leaves and inflorescence rise from the ground, i.e. lacking a visible aboveground stem) or not, or both if populations vary in this trait	whole plant growth form	Binary	Acaulescent (1), non-acaulescent (0), both (2)	2557	100
Erect	Whether palm species has an erect stem (rather than an acaulescent or climbing growth form) or not, or both if local populations vary in this trait	whole plant growth form	Binary	Erect (1), non-erect (0), both (2)	2559	100
StemSolitary	Whether stems are solitary (single-stemmed) or clustered (with several stems), or both if populations vary in this trait	—	Binary	Solitary (1), non-solitary (0), both (2)	2182	85
**Armature**						
StemArmed	Whether bearing some form of spines at the stem or not, or both if populations vary in this trait	—	Binary	Armed (1), non-armed (0)	2502	98
LeavesArmed	Whether bearing some form of spines on the leaves or not, or both if populations vary in this trait	—	Binary	Armed (1), non-armed (0)	2437	95
**Stem size**						
MaxStemHeight_m	Maximum stem height	whole plant height	Continuous	m	2110	83
MaxStemDia_cm	Maximum stem diameter	—	Continuous	cm	1954	76
UnderstoreyCanopy	Understory palms are defined as short-stemmed palms with a maximum stem height ≤5 m or an acaulescent growth form, canopy palms with maximum stem height >5, following classification in ref.^50^	—	Categorical	Understory, canopy, both	2291	90
**Leaf**						
MaxLeafNumber	Maximum number of leaves	—	Count	—	1305	51
Max_Blade_Length_m	Maximum length of the blade (the flat expanded part of a leaf as distinguished from the petiole)	leaf length trait	Continuous	m	1897	74
Max_Rachis_Length_m	Maximum length of the rachis (the axis of the leaf beyond the petiole)	leaf length trait	Continuous	m	1531	60
Max_Petiole_length_m	Maximum length of the petiole (the stalk of the leave)	petiole length	Continuous	m	1209	47
**Fruit**						
AverageFruitLength_cm	Average length of the fruit as provided in a monograph or species description	dispersule length	Continuous	cm	2052	80
MinFruitLength_cm	Minimum fruit length as provided in a monograph or species description	dispersule length	Continuous	cm	906	35
MaxFruitLength_cm	Maximum fruit length as provided in a monograph or species description	dispersule length	Continuous	cm	916	36
AverageFruitWidth_cm	Average width of the fruit as provided in a monograph or species description	dispersule width	Continuous	cm	1993	78
MinFruitWidth_cm	Minimum fruit width as provided in a monograph or species description	dispersule width	Continuous	cm	994	39
MaxFruitWidth_cm	Maximum fruit width as provided in a monograph or species description	dispersule width	Continuous	cm	1002	39
FruitSizeCategorical	Species classified into small-fruited palms (fruits <4 cm in length) and large-fruited palms (fruits ≥4 cm in length), following ref.^50^	—	Categorical	small, large	2052	80
FruitShape	Description of fruit shape as provided in a monograph or species description	—	Categorical	Ellipsoid, elongate, fusiform, globose, ovoid, pyramidal, rounded	1791	70
FruitColorDescription	Verbatim description of fruit color (e.g. red to dark purple, green to orange to red, purple-brown) as provided in a monograph or species description	—	Descriptive	—	1848	72
MainFruitColors	Main fruit colors summarized from fruit color descriptions (black, yellow, orange, red, purple etc.)	—	Descriptive	—	1799	70
Conspicuousness	Main fruit colors classified into conspicuous colors (e.g. orange, red, yellow, pink, crimson, scarlet) vs. cryptic colors (brown, black, green, blue, cream, grey, ivory, straw-coloured, white, purple)	—	Categorical	conspicuous, cryptic	1799	70

The ‘header’ column gives the column names of the trait dataset which is provided as a tab-limited text file^181^. The other information summarizes the description, category of the thesaurus of plant characteristics (TOP)^203^, data type, unit and species coverage information (number and percentage) of the taxonomy and trait data.
